# Association between autism spectrum disorder and peripartum events: a case–control study

**DOI:** 10.1590/1984-0462/2023/41/2021220

**Published:** 2022-07-06

**Authors:** Victor Bruno da Silva, Fernanda Alves Maia, Ana Júlia Soares Oliveira, Ionara Aparecida Mendes Cezar, Laura Vicuna Santos Bandeira, Steffany Lara Nunes Oliveira, Luiz Fernando de Rezende, Vanessa Souza De Araújo Saeger, Marise Fagundes Silveira

**Affiliations:** a Universidade Estadual de Montes Claros, Montes Claros, MG, Brazil.

**Keywords:** Delivery, obstetric, Parturition, Autism spectrum disorder, Parto obstétrico, Parto, Transtorno do espectro autista

## Abstract

**Objective:**

To assess the association between peripartum events and autism spectrum disorder (ASD) development in children and adolescents.

**Methods:**

The current research is a case–control study in northern Minas Gerais state, Brazil. The inclusion criteria in the case group included individuals whose medical records reported an autistic disorder diagnosis, individuals had this diagnosis further confirmed by Northern Minas Autistic Support Association and specialized clinics, and their mothers had to answer positively to the question: “Was your child diagnosed with autism spectrum disorder?” in the data collection instrument. Thus, the case group included 253 mothers of children/adolescents of 2–15 years old diagnosed with autism. The inclusion criteria in the control group included 852 individuals belonging to the same age group and enrolled in the same schools as the case group. A semi-structured questionnaire was applied for mothers of children/adolescents, and the multiple logistic regression model was adopted for data analysis. Gross and adjusted *Odds Ratios* (ORa) were used to estimate the magnitude of the associations.

**Results:**

Autistic disorder was associated with the presence of meconium in amniotic fluid (AF) (ORa 1.67; 95% confidence interval [95%CI] 1.06–2.65) and cesarean delivery type (ORa 1.65; 95%CI 1.17–2.32). Emergency cesarean section increased autistic disorder development likelihood (ORa 2.38; 95%CI 1.61–3.51). Children and adolescents with ASD were more likely to have been exposed to two or more unfavorable peripartum events and obstetric complications than control groups (ORa 1.59; 95%CI 1.01–2.51).

**Conclusions:**

Meconium stained amniotic fluid, delivery by cesarean, and two or more unfavorable peripartum events are variables that should be considered in studies about ASD etiology.

## RESUMO

**Objetivo:**

Avaliar a associação entre os eventos periparto e o desenvolvimento do transtorno do espectro autista (TEA) em crianças/adolescentes.

**Métodos:**

Trata-se de um estudo de caso-controle desenvolvido no norte de Minas Gerais, Brasil. Os critérios de inclusão do grupo caso foram indivíduos com diagnóstico de TEA por laudo médico, confirmado pela Associação Norte Mineira de Apoio ao Autista ou por clínicas especializadas, e as mães que responderam positivamente a: “Seu filho foi diagnosticado com TEA?”. Assim, o grupo caso incluiu 253 mães de crianças/adolescentes com 2–15 anos diagnosticadas com autismo. O critério de inclusão no grupo controle abrangeu 852 indivíduos pertencentes à mesma faixa etária e matriculados nas mesmas escolas que o grupo caso. Um questionário semiestruturado foi aplicado às mães e o modelo de regressão logística múltipla foi adotado para a análise de dados. Para estimar a magnitude das associações, utilizou-se *Odds Ratio* (OR) bruta e ajustada.

**Resultados:**

O TEA foi associado à presença de mecônio no líquido amniótico (ORa 1,67; intervalo de confiança [IC95%] 1,06–2,65) e ao parto cesáreo (ORa 1,65; IC95% 1,17–2,32). A cesárea de emergência aumentou a *chance* de desenvolvimento do autismo (ORa 2,38; IC95% 1,61–3,51). Crianças/adolescentes com autismo apresentaram maior *chance* de exposição a dois ou mais eventos desfavoráveis de parto (ORa 1,59; IC95% 1,01–2,51).

**Conclusões:**

A presença de mecônio no líquido amniótico, cesárea de emergência e mais de um evento de parto desfavorável são fatores que devem ser considerados nos estudos sobre a etiologia do transtorno autístico.

## INTRODUCTION

Autism spectrum disorder (ASD) has become an increasingly prevalent condition and a major public health issue with significant financial, social, and family impacts.^1,2^ ASD is among the top ten causes of disability for 5- to 9-year-old children worldwide.^3^ This neurodevelopmental disorder is characterized by impaired social interaction and communication, in addition to stereotyped and restrictive movement patterns.^1^

According to the latest data from the American Centers for Disease Control and Prevention (CDC), ASD has a prevalence among American children (1 in 54) who aged 8 years with ASD.^2^ In Brazil, data on the prevalence of ASD are scarce or, when available, they are not representative.

Although it is a multifactorial disorder that involves, among its etiological factors, a genetic basis comprising numerous genes and their interaction with the environment, the knowledge about this subject remains unclear.^1,4^ Moreover, incomplete concordance between monozygotic twins and epigenetic mechanisms that explain the effects of environmental factors on gene expression reinforces the contribution of nongenetic factors to the etiology.^4,5^

Peripartum events stand out among factors involved in the pathophysiology of ASD. Pasamanick et al. have conducted the first investigation about this subject and showed, through a case–control study, the link between pregnancy complications and children behavioral disorders.^6^ Since then, several factors associated with peripartum events have been investigated and correlated to ASD development, particularly, changes in amniotic fluid (AF), such as meconium volume and presence,^7–9^ premature rupture of ovular membranes (PROMs),^10^ induced labor,^9^ labor duration,^9–12^ cesarean section,^12–18^ anesthesia use,^14^ and fetal presentation.^9^

Given the scarcity of studies conducted in Latin America and divergences between previous studies, the aim of the present research was to analyze the association between peripartum events and ASD development in children/adolescents.

## METHOD

The current research is part of a larger study entitled “Autism Spectrum Disorder in Montes Claros: a case–control study,” developed in Montes Claros, Minas Gerais, Brazil. The same population was investigated in a recently published study, in which methodological information is described in detail.^19^

To estimate the *Odds Ratio* (OR 1.9^20^ and 0.18) probability of exposure among controls,^21^ we planned the sample size calculation for the independent case–control study. In this study, we planned to analyze several factors associated with ASD; therefore, in the sample size calculation, we considered parameters related to the exposure factor of maternal age of more than 35 years at delivery, for providing the largest sample size among the others tested (i.e., male sex, maternal age, and paternal age). It was also considered a study power of 80%, a significance level of 0.05, and four controls per case. To compensate for possible losses, we increased by 10% and adopted deff=1.5 to correct for the design effect. The required sample size was set at 213 cases and 852 controls.

The subjects who composed the case group were recruited in the Autistic Support of the North of Minas Association (ANDA — *Associação Norte Mineira de Apoio ao Autista*) and in clinics that offer ASD care in the city of Montes Claros (MG). Eight clinics were identified, six with private health services and two with public ones. These institutions were visited and all of them agreed to participate in the research and to disclose names and telephone contacts of 398 mothers of children/teenagers with ASD. The inclusion criteria in the case group were to have an ASD diagnosis confirmed by the researchers based themselves on the criteria proposed by the Diagnostic and Statistical Manual of Mental Disorders (DSM-5). All mothers were contacted by telephone. After three attempts, 304 agreed to schedule a visit for further explanation on the research, and 253 agreed to participate. Thus, the case group included 253 mothers of children/adolescents who aged 2–15 years. To minimize the memory bias, considering that the older the child/adolescent is, the greater the chance of the mother to forget, the child’s/adolescent’s prenatal care card and vaccination booklet were requested to be presented at the moment of the interview. There was 85% agreement between the mothers’ reports and the documents presented.

The control group was composed of neurotypical children and adolescents who did not present the signs of ASD, recruited in 63 regular public, philanthropic, or private schools of Montes Claros. The control group should be in the same age group of the cases (aged 2–5, 6–10, and 11–15 years), with four controls to each case. The children and teenagers were identified by the principals of the schools and their parents were contacted by the researchers in regular school meetings. A total of 1,006 families of children and teenagers were accepted to participate in the research. For ASD screening in the control group, the Modified Checklist for Autism in Toddlers (M-chat) was applied.^22^ Signs of ASD were found in 120 children/teenagers. These children and teenagers were excluded from the research and their mothers were oriented to search for professional help for further investigation. After exclusion criteria were applied, the control group included 886 mothers.

Data collection was individually conducted in person, at a prescheduled time and place, according to the mothers’ availability, between August 2015 and September 2016. A previously trained research team made the appointments and conducted the interviews. A semi-structured instrument was elaborated based on a literature review and reviewed by a multiprofessional team. A pilot study was conducted before data collection.

The peripartum events’ variables analyzed in the current study were as follows: change in AF (presence or absence of oligohydramnios), PROMs (before labor onset and after 20 weeks of pregnancy), induced labor (effective uterine contractions after the use of inducers and/or cesarean section), labor duration (12 h was used as a cutoff point, whereas women who did not show effective contractions, even after attempted induction, were classified as not going into labor), use of predelivery oxytocin (use, or non-use, for labor induction), delivery type (vaginal delivery, elective cesarean section, and emergency cesarean section), use of predelivery anesthesia (use or non-use), fetal presentation (cephalic or non-cephalic), and umbilical cord dystocia, defined as any umbilical cord problems during birth such as circular (presence or absence) and meconium (presence or absence of AF at delivery time). Initial analyses have categorized delivery types as vaginal and cesarean section.

All assessed variables were descriptively analyzed based on their frequency distributions in both groups. The chi-square test was used to assess the association between ASD and other variables; variables presenting significance levels lower than 0.20 (p-value) were subjected to multiple analyses. The stepwise backward logistic regression model was adopted in the multiple analyses; magnitude of the association between outcome and independent variables was estimated through OR at 95% confidence intervals (95%CIs). The number of unfavorable birth events associated with ASD was also evaluated and divided into three groups: hypoxia (presence of meconium in AF and umbilical cord dystocia), changes in AF (PROM and oligohydramnios), and changes in labor and delivery type (fetal presentation, use of predelivery oxytocin, induced delivery, and delivery type).

The analyzed adjustment variables were as follows: child sex (boy or girl), parity (≤2 children and ≥3 children), mother’s age at childbirth (aged <25 years, from 25 to 34 years, ≥35 years), mother’s skin color (self-reported and categorized as white and non-white), socioeconomic class (classes A/B, C, or D/E),^23^ twin pregnancy (presence or absence), family history of ASD (presence or absence), prematurity (gestational age ≥37 weeks or <37 weeks), and crying at birth, defined as the infant’s cry immediately after birth without the need for stimulation (presence or absence). The Hosmer-Lemeshow test and pseudo R^2^ Nagelkerke statistics were used to assess the quality of the adjustment. A correlation matrix between dependent variables was performed, and results did not show multicollinearity between them. All data analyses were conducted using the *Statistical Package for the Social Sciences* (SPSS) statistical software version 23.0 (IBM, Chicago, USA).

The present study followed the ethical precepts defined by the National Health Council for research conducted with human beings, according to resolution 466/2012. The State University of Montes Claros Research Ethics Committee (REC) has approved current research development under opinion number 534.000/14. The legal guardians of all children/adolescents included in the study signed the informed consent form (ICF).

## RESULTS

The final sample included 248 children/adolescents diagnosed with ASD, since 5 cases associated with comorbidities (i.e., two cases of Down syndrome, one case of Rett syndrome, and two cases of fragile X-syndrome) and 886 children/adolescents without signs of ASD were excluded. The groups had similar age ranges, estimated at 6.4 years (standard deviation [SD]=3.6) in the case group and 6.6 years (SD=3.4) in the control group, with minimum age of 2 years and maximum age of 15 years. Table 1 shows the sociodemographic characteristics of the two groups. The case group was made up of 80.0% boys, while the control group was made up of 50.7% boys. ASD group consisted of four times more boys than girls when compared to the control group (p<0.001). The age distribution was uniform between the groups (p=0.132), and most cases and controls aged 2–5 years and 6–10 years, respectively. The groups were also similar according to the type of school they attended (p=0.660), with 67.3% of cases and 64.7% of controls, studying in public or philanthropic schools. As for socioeconomic class, the groups also showed similar distribution (p=0.320), with 60.1 and 56.2% of the families, respectively, of the cases and controls, belonging to social class A or B (Table 1).

**Table 1:** Distribution of cases (n=248) and controls (n=886) according to sociodemographic characteristics. Montes Claros County, MG, Brazil, 2016.

		Case group	Control group	p-value
		n (%)	n (%)	
Sex				
	Male	201 (81.05)	449 (50.68)	<0.001
	Female	47 (18.95)	437 (49.32)	
Age group				
	11–15 years	38 (15.32)	128 (14.45)	0.132
	6–10 years	89 (35.89)	380 (42.89)	
	2–5 years	121(48.79)	378 (42.66)	
School type				
	Private	67 (27.02)	247 (27.88)	0.660
	Not studying	14 (5.65)	66 (7.45)	
	Public/philanthropic	167 (67.33)	573 (64.67)	
Socioeconomic class				
	A or B	149 (60.08)	493 (56.21)	0.320
	C	87 (35.08)	351 (40.02)	
	D or E	12 (4.84)	33 (3.77)	

Based on bivariate analysis, peripartum events’ variables showing association with ASD were preterm birth, presence of oligohydramnios, presence of meconium in AF, non-cephalic fetal presentation, induced labor, prolonged labor and/or not going into labor, anesthesia use, and cesarean delivery type (elective or emergency). All these variables were subjected to multiple analyses (Table 2).

**Table 2:** Characteristics of the case (n=248) and control (n=886) groups based on peripartum events. Montes Claros County, MG, Brazil, 2016.

		Case group n(%)	Control group n(%)	Total* n(%)	ORc (95%CI)	p-value**
Oligohydramnios						
	Yes	21 (8.71)	27 (3.63)	48 (4.88)	2.53 (1.40–4.57)	0.001
	No	220 (91.29)	716 (96.37)	936 (95.12)	1.00	
PROM						
	Yes	22 (9.05)	99 (11.83)	121 (11.20)	0.74 (046–1.21)	0.227
	No	221 (90.95)	738 (88.17)	959 (88.80)	1.00	
Induced labor						
	Yes	125 (50.61)	336 (38.27)	461 (40.98)	1.65 (1.24–2.20)	<0.001
	No	122 (49.39)	542 (61.73)	664 (59.02)	1.00	
Labor duration						
	>12 h	34 (15.04)	70 (8.64)	104 (10.04)	1.71 (1.04–2.68)	0.018
	Did not go into labor	37 (16.37)	194 (23.95)	231 (22.30)	0.67 (0.45–0.99)	0.048
	≤12 h	155 (68.59)	546 (67.41)	701 (67.66)	1.00	
Peripartum oxytocin use						
	Yes	57 (24.46)	219 (26.81)	276 (26.34)	0.88 (0.63–1.23)	0.462
	No	176 (75.54)	598 (73.19)	772 (73.66)	1.00	
Delivery type						
	Elective cesarean section	74 (30.08)	244 (27.82)	318 (28.32)	1.64 (1.16–2.33)	0.005
	Emergency cesarean section	88 (35.77)	178 (20.30)	266 (23.69)	2.68 (1.90–3.78)	<0.001
	Vaginal	84 (34.15)	455 (51.88)	539 (47.99)	1.00	
Anesthesia use						
	Yes	217 (88.57)	692 (79.27)	909 (81.31)	2.03 (1.32–3.10)	0.001
	No	28 (11.43)	181 (20.73)	209 (18.69)	1.00	
Fetal presentation						
	Non-cephalic	39 (15.73)	89 (10.05)	128 (11.29)	1.67 (1.11–2.51)	0.012
	Cephalic	209 (84.27)	797 (89.95)	1006 (88.71)	1.00	
Umbilical cord dystocia						
	Yes	16 (6.61)	58 (6.55)	74 (6.57)	1.00 (0.60–1.80)	0.974
	No	226 (93.39)	827 (93.45)	1053 (93.43)	1.00	
Meconium in the amniotic fluid						
	Yes	47 (18.95)	89 (10.05)	136 (11.99)	2.09 (1.42–3.08)	<0.001
	No	201 (81.05)	797 (89.95)	998 (88.01)	1.00	

Crude *Odds Ratio* and respective confidence intervals.

ORc: Crude *Odds Ratio*; 95%CI: 95% confidence interval; PROM: premature rupture of ovular membrane

* variable associated with *missings*

** chi-square test.

It is worth noting that 34.9% of women who used anesthesia at childbirth did not know which anesthesia was used. Among those who remembered it, similar proportions were observed between the analyzed categories, which recorded approximately 50% of epidural or spinal anesthesia use in both groups.

Based on the multiple analyses, the presence of meconium in AF and emergency cesarean delivery were associated with ASD (Table 3). Based on the analyses in which delivery type was categorized only into vaginal and cesarean deliveries, cesarean delivery also remained significant after adjustments (p=0.004; ORa 1.65; 95%CI 1.17–2.32). However, the cesarean delivery was further categorized into elective and emergency deliveries. It was possible to observe that the magnitude of the association was higher in the group presenting two, or more, unfavorable peripartum events than in the group presenting only one (Table 4).

**Table 3:** Multiple logistic regression analysis of factors associated with autism spectrum disorder. Montes Claros County, MG, Brazil, 2016.

Peripartum events		ORc (95%CI)	ORa (95%CI)	p-value
Presence of meconium in the amniotic fluid				
	Yes	2.09 (1.42–3.08)	1.67 (1.06–2.65)	0.027
	No	1.00	1.00	
Delivery type				
	Elective cesarean section	1.64 (1.16–2.33)	1.24 (0.83–1.87)	0.299
	Emergency cesarean section	2.68 (1.90–3.78)	2.38 (1.61–3.51)	<0.001
	Vaginal	1.00	1.00	

ORc: crude *Odds Ratio*; ORa: adjusted *Odds Ratio*; 95%CI: 95% confidence interval. Model adjusted to child gender, mother’s parity, age and skin color, socioeconomic class, twin pregnancy, family history of ASD, prematurity, and crying at birth. Hosmer-Lemeshow test: p-value=0.333; Pseudo R^2^ de Nagelkerke=0.249.

**Table 4:** Multiple regression model concerning the number of childbirth complications associated with autism spectrum disorder. Montes Claros County, MG, Brazil, 2016.

Number of complications	Case group n (%)	Control group n (%)	ORa (95%CI)*	p-value
1	42 (16.94)	246 (27.77)	0.87 (0.51–1.48)	0.596
≥2	173 (69.76)	451 (50.90)	1.59 (1.01–2.51)	0.045
None	33 (13.30)	189 (21.33)	1.00	

Adjusted *Odds Ratio* and respective 95% confidence intervals.

ORa: adjusted *Odds Ratio*; 95%CI: 95% confidence interval.

* Model adjusted to child gender, mother’s parity, age and skin color, socioeconomic class, twin pregnancy, family history of ASD, prematurity, and crying at birth.

**Figure 1:**
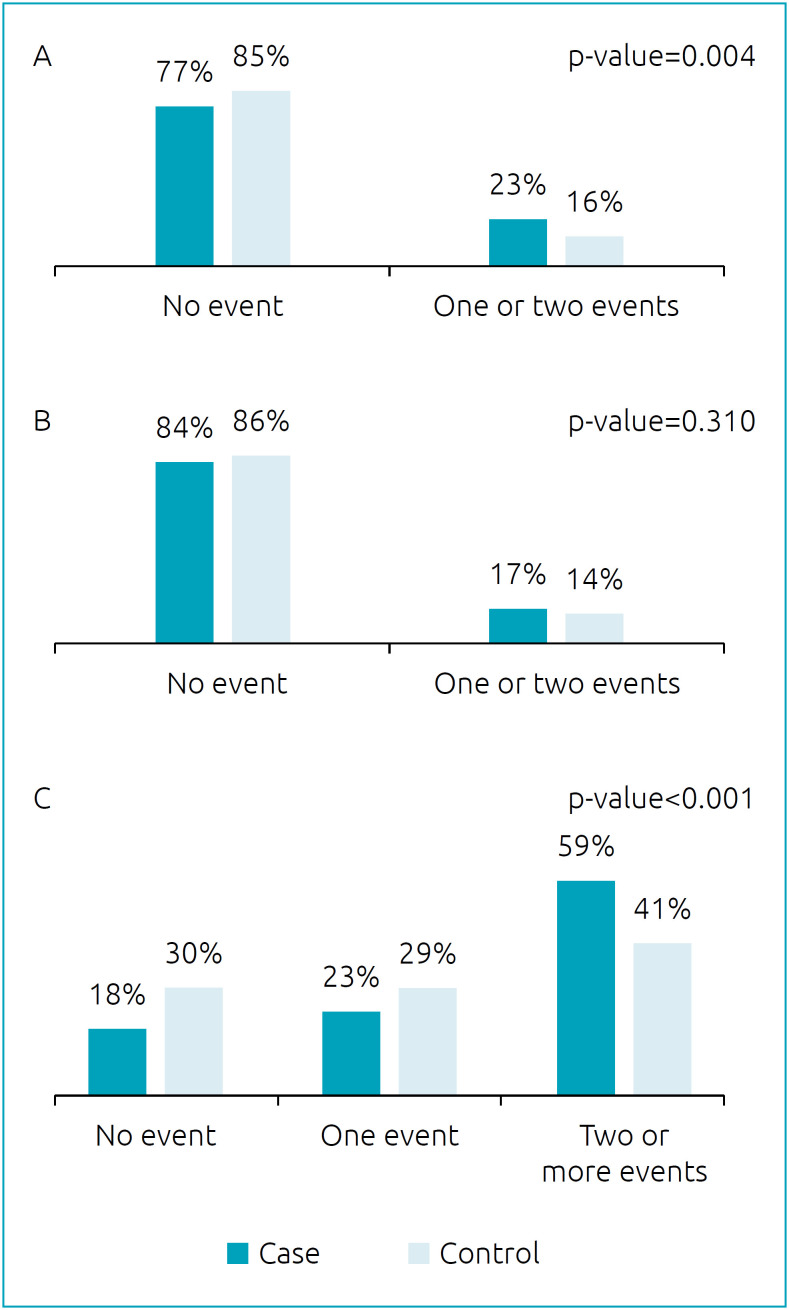
Crude analysis of number of adverse peripartum events associated with autism spectrum disorder, categorized into three groups of unfavorable events. Montes Claros County, MG, Brazil, 2016. (A) Factors related to hypoxia: presence of meconium in amniotic fluid and umbilical cord dystocia. (B) Changes in amniotic fluid: PROM and oligohydramnios. (C) Changes in labor and delivery type: fetal presentation, use predelivery oxytocin, induced delivery, and delivery type.

Based on the categorization of the number of unfavorable peripartum events associated with ASD into three groups, only the group “changes in AF” did not show a statistically significant association with ASD. Groups “hypoxia” and “changes in labor and delivery type” showed a statistically significant association with the disorder (Figure 1).

## DISCUSSION

The presence of meconium in AF and emergency cesarean section have shown an association with ASD in the population investigated in the current study, even after adjustments in genetic (i.e., family members with ASD) and nongenetic factors (i.e., child’s sex, mother’s parity, age and skin color, socioeconomic class, twin pregnancy, family history of ASD, prematurity, and crying at birth). Nongenetic variables associated with ASD development have been increasingly investigated; peripartum events, mainly the ones causing fetal hypoxia, have shown an association with this disorder. It must be highlighted that after grouping of variables that suggest adverse peripartum events, hypoxia (presence of meconium in AF and umbilical cord dystocia), and labor and delivery characteristics (i.e., fetal presentation, use of predelivery oxytocin, induced delivery, and delivery type) showed an association with ASD.

In accordance with previous studies, children/adolescents with ASD were approximately twice as likely to have been exposed to meconium in the AF as the ones in the control group.^7,8^ According to Miller et al. (2017), children exposed to meconium were more likely to be diagnosed with ASD than nonexposed children.^7^ Increased risk of ASD was also identified in the adjusted analyses.^7,8^ Such association between ASD and meconium aspiration by babies was not evidenced in the meta-analysis performed by Gardener et al. and in the cohort study conducted by Miller et al.^7,9^ It is worth noting that exposure to meconium does not mean aspiration.^7,9^ It was not possible to investigate whether children/adolescents assessed in the present study presented meconium aspiration syndrome.

Meconium (feces resulting from undigested waste) is often eliminated soon after childbirth.^7^ However, meconium released during the intrauterine period may be associated with incidence of fetal stressors such as hypoxia.^9,24^ Thus, it is likely that meconium exposure itself is not the one causing neural damage but a common stressor that influences both meconium release and neurodevelopment.^7^ Hypoxia can cause cellular trauma and trigger meconium release; however, the absence of respiratory distress may hinder neonatal hypoxia detection and treatment, a fact that may lead to some neurological impairment level, as seen in ASD.^7^ Thus, events leading to fetal hypoxia have been identified as a common mechanism for several ASD risk factors.^10,25^ In addition, individuals diagnosed with ASD tend to have complications during pregnancy, which often involve situations that lead to fetal hypoxia.^25^ Fetal hypoxia is associated with increased dopaminergic activity, which was already associated with ASD.^10,26^

In addition to the presence of meconium in the AF, other factors indicating hypoxia indicate preterm birth, birth weight, fetal distress, absence of crying at birth, PROMs, and umbilical cord dystocia — they are oxygen deprivation factors that can cause brain damage.^27^ PROMs and umbilical cord dystocia were not associated with ASD, based on the gross analysis conducted in the present study, while absence of crying at birth has shown an association with ASD, after confounding factor adjustment, in a previously published study conducted with this very same population.^19^ Despite the presence of meconium and umbilical cord dystocia are related to fetal hypoxia, it is not possible to state that these outcomes always indicate hypoxia, especially when the problem is promptly identified and treated in time.

Delivery type is another factor that has been associated with increased likelihood of ASD development. The children/adolescents with ASD investigated in the current study presented higher likelihood of being born by cesarean section than children/adolescents without ASD. However, based on the multiple analyses conducted, cesarean section was categorized into elective and emergency cesarean deliveries, where only emergency cesarean section maintained a significant association with ASD. This outcome has evidenced that children/adolescents with ASD were twice as likely to have been born by emergency cesarean section.

Elective and emergency cesarean deliveries are characterized by different factors; therefore, they may be associated with neurodevelopment in a different way.^18^ Emergency cesarean sections are often performed due to some complications.^11,25^ Thus, ASD may not be associated with the surgical procedure itself but with factors that led to it.^13,18^

However, despite being a life-saving procedure in the case of possible complications, there is no evidence that cesarean delivery performed without indication is beneficial to the child.^18^ On the contrary, cesarean section is associated with a number of short- and long-term health issues such as neurodevelopmental impairment.^18^ Several hypotheses have attempted to explain the association between cesarean delivery and ASD, such as oxytocin dysregulation, microbiota-gut-brain axis, and nervous system toxicity caused by anesthesia application during cesarean sections.^16^ Thus, the fact that the number of cesarean deliveries has increased considerably in recent years has raised significant concern. According to estimates, cesarean deliveries account for approximately 20% of all childbirths worldwide; they range from 7% in Africa to 41% in Latin America and the Caribbean.^28^ This representation is even higher in Brazil, where cesarean sections account for almost 50% of all childbirths; this index is approximately three times higher than that recommended by the World Health Organization.^29^ This trend is likely due to medical professionals and pregnant women’s preferences rather than to adverse clinical conditions.^29,30^

Other peripartum events, such as the incidence of oligohydramnios, induced labor, labor duration, anesthesia use, and fetal presentation, are also associated with delivery type; these events remained associated with ASD in the present study — based on the bivariate analysis — and lost significance after adjustments. This outcome draws attention to other factors, whether they are genetic or not, which may be behind this association.

With respect to the number of unfavorable peripartum events, the group presenting two, or more, unfavorable events had a positive association with ASD. These results are consistent with previous studies, which showed that children with ASD were more likely to have had at least one unfavorable event during pregnancy and/or childbirth than their neurotypical siblings^30^ as well as that the incidence of any type of complication was higher in the group of children with ASD than in the group of children without ASD.^18^ These data reinforce the importance of monitoring individuals who have had at least one unfavorable event during childbirth in order to help identify signs of ASD and to facilitate its early diagnosis.

The current study had some limitations: data source was based on mothers’ reports, which may have been subject to memory bias; however, there was consistency between the mothers’ reports and the documents presented; ASD diagnosis was performed by different teams; the impossibility to determine the indications for emergency cesarean sections and to confirm whether or not they were elective based on the analysis of medical records; and the impossibility to confirm whether the individuals included in the study had meconium aspiration syndrome. It is worth noting that an adaptation of the use of the M-CHAT screening instrument was carried out beyond the expected age range (mothers oriented to answer about the children’s characteristics during the expected age range).

Although the model estimated to evaluate the association of perinatal factors and ASD was adjusted by a series of confounding variables, such as child gender, mother’s parity, age and skin color, socioeconomic class, twin pregnancy, family history of ASD, prematurity, and crying at birth, this model should still be interpreted with caution, since there are other factors correlated to ASD discussed in the literature that were not considered in this study.

A causal relationship was not the object of this work and also cannot be established with the data from the present study because of the design used and also because it does not consider some underlying diseases that have hypoxia-related variables as consequences. It is worth noting that in the hypoxia group, it is not possible to precisely define its occurrence; however, given the information available in the literature, it is possible to assume its occurrence under the conditions described in the hypoxia group.

However, this is, to the best of our knowledge, the first study to address ASD and childbirth events in Latin America, based on a sample size of this magnitude (248 cases and 886 controls). In addition, analyses were adjusted for several factors known to be associated with ASD that may influence childbirth events, and the diagnosis of individuals belonging to the case group was not only based on reports but further confirmed by qualified professionals.

Peripartum events that emerged as significant risk factors for ASD in the present study, such as the incidence of meconium in the AF and emergency cesarean delivery, have suggested that fetal hypoxia is likely to be an important factor for ASD development. These findings have shown the complexity of factors associated with childbirth events in ASD etiology. Results in the current study have also indicated that children/adolescents with ASD were more likely to have been exposed to two, or more, unfavorable peripartum events.

It is worth emphasizing that unfavorable peripartum events are preventable and changeable and that understanding these factors is important to help prevent ASD and to support the development of public policies focused on actions aimed at favoring its diagnosis and immediate intervention and, consequently, at improving the prognosis of individuals with ASD, at supporting their family members, and at reducing public spending on this disorder.

ASD is a complex, multifactorial disorder, which has entailed several factors in its etiology. Studies that address perinatal variables, preferably with a prospective cohort design, may better clarify the causal relationship between these factors and ASD.
